# Supplementary material for: Splice-Junction-Based Mapping of Alternative Isoforms in the Human Proteome
Source: Cell Rep. Author manuscript; Available in PMC 2020 Jan 15. (PMC6961840; doi:10.1016/j.celrep.2019.11.026)

A

sp|P07919|QCR6_HUMAN|ENSG00000173660|MXE2|2531|chr1|46303820|46309127|+0|r1566|T1
MLTESGDPEEDPLTTVR q value: 2.9253e−05 Tr_novel:TRUE RefSeq_Novel:FALSE
Search result spec prec mz: 945.4503 Actual spec prec mz: 945.45035
Fragments matched per AA: 1.47 Proportion of top 20 peaks matched: 0.5

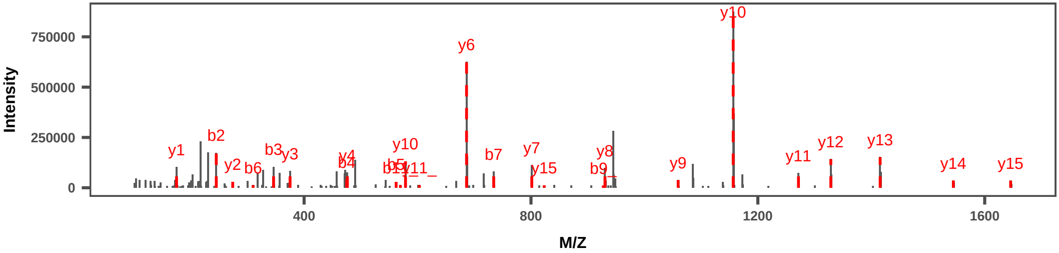

B

Scatterplot of predicted elution time
Fitting R2: 0.893
Novel peptide residual Z score: 1.24
Number of peptides: 1742

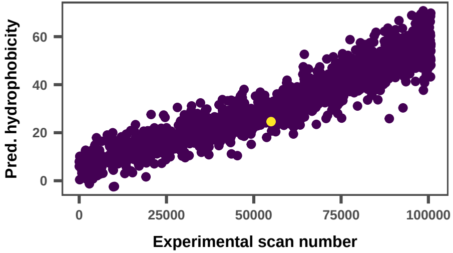

C

Distributions of residuals from best−fit line
of predicted RT vs Expt. scan number
Line: Z score of novel peptide
Z: 1.24

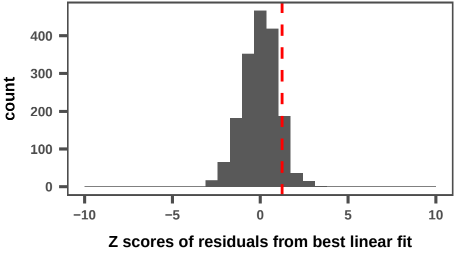

Supplement: 2 [file NIHMS1546469-supplement-2.zip › DF1/PXD006675/RightAtrium/RightAtrium_16_UQCRH_MLTESGDPEEDPLTTVR.pdf]
